# Design and implementation of *#Talking about School Transitions 5-7*; a longitudinal skills-based primary-secondary school transitions curriculum

**DOI:** 10.3389/fpsyg.2026.1864509

**Published:** 2026-08-18

**Authors:** Charlotte Louise Bagnall, Elizabeth Stevenson

**Affiliations:** 1 Manchester Institute of Education, The University of Manchester, Manchester, United Kingdom; 2 St. Paul’s Community Development Trust, Birmingham, United Kingdom

**Keywords:** emotional wellbeing, intervention science, knowledge mobilization, mental health, primary-secondary school transitions

## Abstract

Primary-secondary school transitions are emotionally demanding, critical periods for two-thirds of children, who report stress and anxiety up to 2 years before and after starting secondary school. Maladjustment is associated with poor attainment, attendance and mental health difficulties, especially for more disadvantaged groups. Thus, from a public health perspective, timely support is crucial, which is recognised in policy, practice and research. However, significant limitations pervade all three sectors, with primary-secondary school transition consistently noted as “a period not handled well” (March, p. 65), and interventions “do not give enough importance to improving emotional wellbeing and how schools might be supported in this role”. Existing interventions are limited in focus, often targeting social adjustment and academic attainment rather than emotional wellbeing; reach, with few programmes spanning three school years; sustainability, due to minimal stakeholder involvement; and rigorous evaluation. The present paper aims to address these limitations, by presenting the theory, empirical research, and methodological decisions, informing the design and implementation of *#TaST 5-7*: the first emotional-centered, skills-based intervention, delivered contiguously across Year 5, 6 and 7. This research makes a unique methodological contribution through presenting a worked-example of integrative co-production and rigorous component analysis. It advances empirical understanding of how to best support children’s emotional wellbeing in the context of primary–secondary transitions through a theoretically grounded intervention structure, builds practitioner capacity for evidence-informed support by presenting an outline of the *#TaST 5-7* lessons and offers a replicable model for future intervention design, implementation, and evaluation.

## Introduction

Primary-secondary school transitions are widely acknowledged as critical periods for children. While many children feel optimistic about the new opportunities they will have at secondary school, a substantive body of research also shows that this time is often accompanied by feelings of stress, anxiety, and declines in emotional wellbeing (Rice et al., 2021), which can begin approximately two years prior (Bagnall et al., 2024a) and can continue two years into secondary school (Jindal-Snape and Cantali, 2019). Poor experiences of primary-secondary school transitions are associated with poor educational attainment, attendance, social adjustment and mental health, especially for more disadvantaged groups (Donaldson et al., 2025). From a public health perspective, primary-secondary school transitions presents a critical entrance point for early-intervention support that promotes children’s emotional wellbeing, which is recognised by parents/carers (Bagnall et al., 2020), educational practitioners (Bagnall et al., 2025a), by children themselves (Garner and Bagnall, 2024), and within educational policy (Children’s Commissioner, 2024).

However, to date the corpus of existing primary-secondary school transitions interventions are limited in (1) foci (e.g., existing programmes have focused on children’s social adjustment and academic attainment, which can be more easily targeted, with a lack of focus on children’s emotional wellbeing); (2) reach (e.g., lack of early-intervention contiguous programmes, spanning three school years, and untested assumptions about the necessity of both pre- and post-transition support); (3) sustainability (e.g., few interventions consider ecological fit and social validity, by neglecting stakeholder involvement); and (4) rigorous evaluation methods (e.g., imprecise longitudinal evaluations in terms of timing and level comparisons drawn, and the absence of early baseline data collection) (Beatson et al., 2023). The present paper aims to address these limitations, through presenting the theory, empirical research, and methodological decisions, which informed the design and implementation of *#TaST 5-7*: the first emotional-centered, skills-based intervention, that is delivered contiguously across Year 5, 6 and 7.

In doing so, the present research, makes a unique methodological contribution through providing a worked-example of integrative co-production, drawing on rigorous component analysis, and iterative stakeholder consultation. This was paramount to ensure that innovation was evidence-informed and grounded in established mechanisms of change, but also practice-led to maximise effectiveness, confidence in scalability, and transferability across diverse school settings, directly tackling ongoing limitations that pervade the sector. This offers significant empirical and theoretical advancements in refining existing interventions and improving the design, implementation, and evaluation of future initiatives. Furthermore, by presenting the theory and empirical evidence underpinning *#TaST 5–7*, the present study demonstrates practical utility by equipping educators with the knowledge to understand children’s emotional needs in the context of primary–secondary transitions and build capacity for implementation of evidence-informed strategies to best support them.

## Background

There is a growing body of research with a clear shared contemporary conceptualisation of educational transitions, as a multi-dimensional ongoing process which spans across multiple domains and contexts (Jindal-Snape et al., 2021). This is theorised in Jindal-Snape’s (2016, 2023) *Multiple and Multi-dimensional Transitions Theory (MMT)*, which conceptualises the changes children negotiate during educational transitions, such as primary-secondary school transitions, as “transitions” in their identity (e.g., primary/secondary school child, child/young person); relationships (e.g., friendship groups and teacher support), formal (e.g., school size and structure) and informal (e.g., climate and ethos) school environment; teaching styles and academic expectations. These transitions occur at the same time, over time, in multiple domains (e.g., social, academic), across multiple contexts (e.g., school, home) and are multi-layered (e.g., child’s parents/carers and peers are also navigating primary-secondary school transitions which may interact with or instigate other transitions for the child). Primary-secondary school transitions can also coincide with the biological (e.g., puberty), social (e.g., peer influence) and psychological (e.g., identity formation) demands of early adolescent development, which is marked by cognitive and emotional brain development (Blakemore and Mills, 2014). Furthermore, in England, primary-secondary school transitions are also nested amongst school-based pressures, such as school choice decisions and academic national Standard Assessment Tests, which can further disrupt children’s cognitive and social processing and perpetuate feelings of instability (Bharara, 2020).

Unsurprisingly, negotiating multiple changes over primary-secondary school transitions, can be emotionally demanding, and heavily draw on children’s ability to cope (Curson et al., 2019; Donaldson et al., 2023). For example, research has shown that primary-secondary school transitions are almost always accompanied by concerns for children, their parents and teachers (Rice et al., 2021), which can begin from as early as Year 5 (two years prior to primary-secondary school transitions in England) (Bagnall et al., 2024b) and can continue two years into secondary school (Jindal-Snape and Cantali, 2019). This is often due to a mismatch between the anxiety children experience during primary-secondary transition and the emotional skills they can draw on to cope, with many children discussing the importance, but also difficulty in managing their emotions over primary-secondary school transitions (Bagnall, 2020). Furthermore, when children are asked to reflect on their primary-secondary school transitions, many children report feeling that they had underestimated the importance of the socio-emotional aspects of their transitions when in primary school, and as a result feel insufficiently prepared for transfer challenges (Bagnall et al., 2021a).

If prolonged, and support is not provided, children who experience poor emotional wellbeing following primary-secondary school transitions, are concurrently and prospectively more at risk of experiencing educational disruption (including attainment), social maladjustment, and poor mental health (Demkowicz et al., 2023; White, 2020). These outcomes can contribute to poor life chances and exacerbate existing social inequalities, especially for more vulnerable children, such as those with special educational needs (Bagnall et al., 2021b; Kelly et al., 2026), in receipt of Pupil Premium Funding (Garner and Bagnall, 2024; Moore et al., 2020) and have been or are at risk of being excluded and/or suspended (Dunnett et al., 2025). Thus, supporting children’s emotional wellbeing, over primary-secondary school transitions is a significant public health issue. This is recognised by key stakeholders, including children, parents and educational practitioners; all of whom highlight the importance of emotional-centered discussions during this time (Bagnall et al., 2020).

However, within practice, the multiple and disparate challenges that primary-secondary school transitions present can be difficult to manage, particularly in the absence of clear, consistent and evidence-informed guidance, regarding what to prioritise, how to intervene effectively, and when (Bagnall et al., 2026). Ensuring that primary-secondary school transitions provision is evidence-informed, co-produced with educators and accessible is therefore vital. While academically developed and endorsed primary-secondary school transitions interventions efficacious, feasible to deliver, and suitable for universal and targeted populations within schools, have been synthesised and evaluated in academic literature; these evidence-based recommendations are not being accessed nor implemented by educators (Bagnall et al., 2024a). Instead, practitioners frequently rely on anecdotal sources to inform practice, resulting in “patchwork practice” across schools and Local Authorities and leading to inconsistencies in the quality and scope of support offered (Growney, 2022). Given the constraints on schools’ time and financial resources, educational practitioners require access to high-quality, evidence-informed approaches to primary–secondary school transitions. It is therefore essential that researchers work collaboratively with practitioners through co-production to ensure that transitions practices are both evidence-informed and grounded in real-world practice (Bagnall and Jindal-Snape, 2023), which the present research seeks to achieve.

This need is further reflected in educational policy, with Government reports in England identifying primary–secondary transition as a period “not handled well” (Ofsted, 2015b, March, p. 65), with many children believed to be “lost in transition” (Children’s Commissioner, 2024, p. 96). It has also been recognised that current transition interventions “do not give enough importance to improving resilience and well-being and how schools and colleges might be supported in this role” (Department for Education, 2018, p.13). The importance of supporting children’s emotional wellbeing over primary-secondary school transitions has been further intensified by the international outbreak of COVID-19, which has shown the negative impact of primary-secondary school transitions on children’s emotional wellbeing to heighten (Bagnall et al., 2022), and there is evidence to suggest that schools are still recovering, especially in the context of supporting children’s resilience and coping skills (Garner and Bagnall, 2024).

Primary-secondary school transitions are therefore increasingly recognised in research, policy and practice as periods of heightened sensitivity to both adaptive and maladaptive change, offering a critical opportunity to implement preventative early-intervention support. Schools’ wide reach, prolonged period of engagement, and central role in communities provide an ideal setting and delivery-system (since most children, including those from marginalised groups, spend a large proportion of their day at school) to implement universal interventions that focus on supporting children’s emotional wellbeing and preventing the development, maintenance, and escalation of mental health difficulties (O’Brien et al., 2023). School-based interventions are also perceived as less threatening (by parents and children), can influence outcomes for children who would not otherwise access support through usual care pathways; and shown to be more cost-effective than targeted approaches, which is important given schools’ stretched time and financial resources to address preventative mental health concerns (Department for Education, 2023a; Welsh Government, 2022). This can be evidenced by the many formal induction programmes implemented by primary and secondary schools to improve children’s adjustment during this period (Neal et al., 2016).

However, the strength of evidence differs considerably, which in part might be shaped by variations in the design (top-down vs. bottom-up approach), inclusion criteria (universal, targeted and proportionate universalism) and breadth of foci of interventions. For example, methodologically sound and theoretically supported interventions imposed on schools with little consultation have been shown consistently to impede sustainability (Stormshak et al., 2016), particularly given schools’ competing priorities, limited time and financial resources (Bonell et al., 2015; Trotman et al., 2015). Similarly, interventions taking bottom-up approaches are leading to a plethora of reactive and largely not evidence-based programmes, leading to context-sensitive patchwork practice. This can limit synthesis, comparison and integration of evidence across studies and settings to avoid data fragmentation, and uncertainty pertaining to what works to bring about change. Thus, to avoid short-term, unsustainable programmes, ongoing evaluation and refinement is needed, drawing on iterative stakeholder co-production; a significant strength of the present research to ensure alignment between underpinning research and theory, with real-world practice.

In part these limitations are shaped by lack of consistency in (a) the outcomes interventions target, with most interventions focussed on symptomology as an outcome (as opposed to developing children’s skills), taking a curative, as opposed to a preventative approach; and (b) the psychometrics used. Overcoming these limitations Bagnall et al. (2024b) have designed and validated the *Primary-Secondary School Transitions Emotional Wellbeing Scale* (*#P-S WELLS*) which comprehensively measures children’s emotional wellbeing in the context of primary-secondary school transitions, identifying which aspects of moving to secondary school children are experiencing emotional difficulties with, who might be particularly vulnerable and what support could be useful. However, to realise a national initiative in supporting children’s emotional wellbeing in the context of primary-secondary school transitions, work is not only needed to identify a feasible measurement strategy, consisting of termly monitoring to identify children’s needs (Bagnall et al., 2025b), but also to respond to these needs through evidence-informed intervention. The present research addresses this gap, by outlining the design and implementation of *#TaST 5-7*: the first emotional-centered, skills-based intervention, that is delivered contiguously across Year 5, 6 and 7.

### Rationale

Responding to longstanding calls to strengthen conceptual clarity, methodological decision making and ecological fit within the design and implementation of school-based interventions, the present research provides a worked-example of the theory, research and methodological decision-making, which informed the design and implementation of *Talking about School Transitions 5-7* (*#TaST 5-7*). The design of *#TaST 5-7* was informed by Beatson et al.’s (2023) systematic review, which synthesised and evaluated evidence to date pertaining to the design, selection, implementation and evaluation of primary-secondary transition interventions. The following key limitations were identified: (1) lack of emotional wellbeing foci; (2) reach (e.g., lack of early-intervention contiguous programmes, spanning three school years, and untested assumptions about the necessity of both pre- and post-transition support); (3) sustainability (e.g., few interventions consider ecological fit and social validity, by neglecting stakeholder involvement); and (4) rigorous evaluation methods (e.g., imprecise longitudinal evaluations in terms of timing and level comparisons drawn, and the absence of early baseline data collection), which *#TaST 5-7* directly narrows. Drawing on these limitations, an integrative co-production approach is taken, drawing on rigorous component analysis, and iterative stakeholder consultation, to ensure that innovation is evidence-informed and grounded in established mechanisms of change, but also practice-led to maximise effectiveness, confidence in scalability, and transferability across diverse school settings. Please note, that the evaluation of the intervention will be reported in a separate publication.

#### *#Talking about School Transitions 5-7* (*#TaST 5-7*)

**FIGURE 1:**
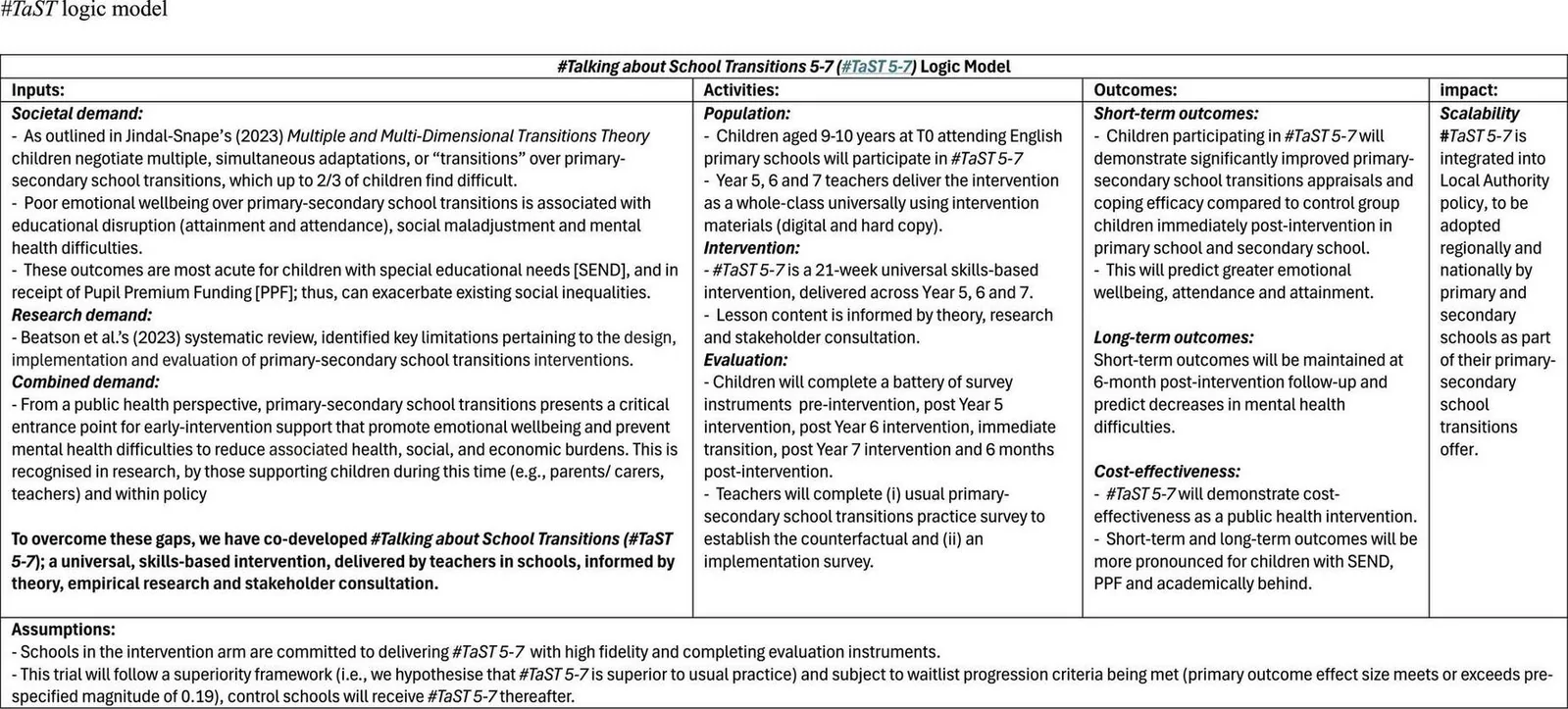
*#Talking about School Transition* (*#TaST*) logic model. This work is licensed under the Creative Commons Attribution-NonCommercial- NoDerivatives 4.0 International License. To view a copy of this license visit: www.creativecommons.org/licenses/by-nc-nd/4.0.

*#Talking about School Transitions 5-7* (*#TaST 5-7*) is a 21-week universal, skills-based transition curriculum, which is delivered by teachers in schools. See Table 1 for a synthetic table presenting the detailed structure of the *#Talking about School Transition 5-7* (*#TaST 5-7*) curriculum. *#TaST 5-7* takes an early-intervention approach, through developmentally sequenced lessons delivered across the last two years of primary school (Year 5 and 6) and first year of secondary school (Year 7), to gradually develop children’s awareness, knowledge and ability to cope with the multiple changes they will experience over primary-secondary school transitions, through scaffolding a repertoire of socio-emotional skills. In doing so *#TaST 5-7* aims to improve children’s primary-secondary school transitions appraisals and coping efficacy, to ultimately improve their emotional wellbeing, and reduce mental health difficulties. These hypothesised mechanisms are articulated in our logic model (see Figure 1), which was developed in partnership with educational practitioners to explicitly outline how and why *#TaST 5-7* is anticipated to lead to the desired outcomes (Humphrey et al., 2016).

**TABLE 1:** Synthetic table presenting the detailed structure of the *#Talking about School Transition 5-7* (*#TaST 5-7*) curriculum.

Lesson title	Date	Key aim	Brief description of lesson content; and example activity
Lesson 1: asking for help	Year 5: April	To develop pupils’ confidence and equip them with the practical skills needed for seeking help - a foundation for navigating the challenges of primary-secondary school transitions.	This session will help pupils develop the skill of asking for help when it is needed, especially from people (adults and children) that they don’t know or trust yet. **“Hello, I am” activity:** pupils take on the role of different types of teachers and practice introducing themselves to each other, experimenting with talking to unfamiliar people in a low-pressure, drama-based setting. **“Please sir” activity:** in pairs, one pupil acts as the teacher and the other as a pupil, coming up with questions to ask each other and recounting advice to the rest of the class.
Lesson 2: identifying independence	Year 5: May	To develop the organisational, self-management, and autonomous decision-making skills that will support pupils in meeting the increased expectations of secondary school.	Helping pupils recognise the times they are/know how to be independent. This is recognising that independence looks very different between primary and secondary school. **Along the line activity:** pupils position themselves on a continuum from 100% support to 100% independence in response to simple tasks to more complex ones, prompting discussion about developing independence at secondary school. **Pack your school bag workbook activity:** pupils use a timetable to identify what items they would need on given school days, and pack their paper school bag, building organisational skills.
Lesson 3: progression vs. loss	Year 5: June	To help pupils identify, articulate, and make sense of the conflicting emotions that can accompany primary-secondary school transitions, and doing so supporting their emotional awareness, understanding and regulation.	This session will help pupils to start thinking about primary-secondary school transitions as a progression, by balancing feelings of loss toward leaving primary school with anticipation for the new experiences they will encounter at secondary school. **Primary school progression workbook activity:** pupils independently reflect on their time at primary school, including achievements and things they have learnt, and consider how this prepares them for secondary school, drawing on the positive psychology principle that recalling positive experiences promotes positive behavior. **Life Transitions workbook activity:** pupils reflect on a previous transition experience inside or outside of school and consider how it can help prepare them for primary-secondary school transitions.
Lesson 4: choices/decisions	Year 5: July	To equip pupils with the tools and skills needed to make thoughtful, adaptive choices during the secondary school selection process and beyond.	This session will support pupils in identifying when and how they make their own decisions and scaffolding skills to continue doing this in primary and secondary school. **Choosing secondary school workbook activity:** pupils draw up a list of what they feel is important in a secondary school, prioritise their criteria, and consider how to find out more information, developing a framework for decision-making. **IT activity:** pupils pair their priorities from the worksheet to actual secondary schools in the local area to help inform their school choices.
Lesson 5: expectations	Year 6: September	To develop a realistic understanding of the social, academic, and environmental changes ahead, so pupils feel prepared for secondary school.	This session will look at pupils’ expectations of secondary school, managing expectations and dispelling the myths! **“The Good, the Bad and The Ugly!” workbook activity:** following a class discussion, pupils complete a worksheet categorising their feelings about secondary school into things they are excited about (Good), a little worried about (Bad), and terrified about (Ugly), using gesture and exaggeration to lighten the mood. **Forum Theater activity:** in pairs, pupils take on teacher/pupil roles and act out one Bad/Ugly situation, with the audience suggesting and enacting solutions, exploring different ways of resolving anticipated challenges.
Lesson 6: coping with change	Year 6: October	To help pupils explore and constructively address their thoughts, feelings, and behaviors in response to the multiple changes associated with primary-secondary school transitions.	This session will help pupils to develop coping strategies to overcome challenges they may experience over primary-secondary school transitions. **Transition similarities and differences workbook activity:** groups rotate around four tables labeled with social, academic, practical, and emotional domains, using green pens to note similarities and red pens to note differences between primary and secondary school, before selecting two to record in their workbook. **Change and solutions workbook activity:** pupils independently take an Ugly situation from Lesson 5 and match it with a positive thought, practicing reframing as a coping strategy.
Lesson 7: navigating	Year 6: November	To develop practical navigation skills including: map reading, following instructions, and spatial awareness, to reduce anxiety around moving through an unfamiliar secondary school environment.	This session will look at navigating around new surroundings, including understanding timetables and systems. **“To you… To me” workbook activity:** in pairs using local area maps, pupils take turns giving instructions to get from point A to point B, practicing going to a destination and back, developing directional language and spatial reasoning. **Route writer workbook activity:** pupils draw routes between two points on multi-level school maps and write directions, then work in pairs to guide each other along routes, repeating the exercise to explore how repetition supports memory and preparation.
Lesson 8: structure/life changes	Year 6: December	To support pupils in understanding and adapting to the structural and identity changes that accompany primary-secondary school transitions, both at home and in school.	This session will look at how life at secondary school will be different, including new routines before and after school. **“Well, I never” activity:** using post-it notes or whiteboards, pupils identify things that will be different at secondary school and for each one indicate whether they feel prepared and confident, expect it but don’t know what to do, or are surprised by it, building realistic awareness and coping strategies.
Lesson 9: making friends	Year 6: January	To promote peer relationship skills and facilitate positive connections, recognising that strong peer support is a key predictor of successful transitions adjustment.	This session focuses on how to make and manage friendships; a skill that many pupils have not had to do since starting school. **The co-pilot activity workbook activity:** pupils first independently complete a personal pledge about how they will prepare for secondary school, then pair with a classmate (ideally transitioning to the same school) to discuss how they can support each other, before writing a co-pilot pledge in their partner’s workbook. **“No one told me” activity:** using Forum Theater, pupils role play different ways of initiating conversations and making friends, modeling and suggesting strategies for striking up connections in unfamiliar social situations.
Lesson 10: parent/carer support	Year 6: February	To highlight the role of parents/carers as a vital source of continuity and support during primary-secondary school transitions, and to help pupils actively draw on their support.	This session focuses on encouraging open communication and discussion channels between parents/carers and children in the lead up to primary-secondary school transitions. **Parent/carer puzzle workbook activity:** pupils independently write eight questions they would like to ask a parent/carer or older family member about primary-secondary school transitions, transfer these onto a cut-out puzzle, and take it home to interview a family member, noting their interviewee’s responses in their workbook.
Lesson 11: teacher support	Year 6: March	To help pupils understand how teacher-pupil relationships differ at secondary school and develop strategies for continuing to access teacher support in the new environment.	This session helps pupils to recognise that they can and should still access support from teachers when they move to secondary school, although the way they do this may change, reflective of changes within the secondary school environment.
			**“Same old same old” activity:** in pairs, pupils create and repeat teacher/pupil scenes with different types of teachers, focusing on how responses and communication styles vary, and discussing which teacher is the right one to approach with different concerns.
Lesson 12: WoW	Year 6: April	To consolidate and celebrate the skills and strategies pupils have developed throughout the *#TaST 5-7* curriculum, building confidence and resilience as they approach primary-secondary school transitions.	This is a reflective session working with pupils to remind them of what they have learnt over the *#TaST 5-7* curriculum, whether their thoughts, feelings and emotions around starting secondary school have changed and how to continue managing them. **“I was BUT now I am” activity:** in small groups using hot seating, pupils highlight at least one thing they were previously worried about regarding secondary school and how they feel about it now, modeling the skills they have developed. **“I am feeling” activity:** pupils add a word or picture to an A1 sheet under Good/Bad/Ugly headings to capture how they currently feel about secondary school, with the class together exploring the balance of positive and negative feelings as a celebration of progress.
Lesson 13: cross-aged teaching	Year 6: June	To deepen pupils’ own learning by teaching younger peers, reinforcing their knowledge and skills through the process of explanation, reflection, and connection-making.	This session helps pupils to consolidate their learning from the *#TaST 5-7* curriculum by using cross-aged teaching techniques. **Transition top tips workbook activity:** pupils independently reflect on challenges they may face at secondary school and how to overcome them, generating their five top tips for a successful transition. Pupils then work independently, in pairs, or in groups of four to turn their top tips into a poster, leaflet, poem, storyboard and/or role play, which they then present to Year 5 pupils.
Lesson 14: asking for help	Year 7: September	To build pupils’ confidence in seeking help in their new secondary school environment, recognising that this skill is essential for fostering independence, resilience, academic success, and wellbeing.	This activity will help pupils to develop the confidence in asking for help in secondary school, by scaffolding pupils’ confidence in speaking to adults and children that they don’t know or trust yet. **“This or That” activity:** pupils share likes and dislikes through a game to practice sharing information and build new relationships with peers and staff. **“What if” activity:** pupils anonymously write questions about secondary school, which are drawn at random and answered by the class, building confidence in asking for help in a safe, non-judgmental space.
Lesson 15: navigating	Year 7: September	To reduce anxiety around getting lost by equipping pupils with the practical skills to read a school map and navigate confidently between places in their new school building.	This activity encourages pupils to get to know, and navigate between, places in their new secondary school building, by learning how to read a school map. **Secret Maps workbook activity:** working in pairs, one pupil draws a route between two points on the school map and describes it to their partner without showing them. The partner identifies where they have ended up, prompting discussion about what went well or wrong. Extension: pupils plan and complete a real journey at home or in school.
Lesson 16: getting to know other pupils	Year 7: September	To support friendship building and peer connection by providing structured opportunities for pupils to get to know their classmates in a safe and inclusive way.	This activity supports pupils in getting to know other pupils in their class, to support friendship building and peer support. **People Bingo activity:** set to an upbeat song, pupils move around the room to find classmates who match descriptions on their bingo card, adding names to boxes rather than simply crossing them off, to help learn each other’s names. Extension: pupils create their own themed bingo cards as the year progresses.
Lesson 17: speaking to new teachers	Year 7: September	To develop pupils’ confidence in communicating with new teachers and understanding how to adapt their communication style appropriately depending on who they are speaking to.	This activity focuses on developing pupils’ confidence in speaking to new secondary school teachers, and the skill of finding and being an appropriate version of themselves based on who they are speaking to. **The appropriate me workbook activity:** pupils list new people they have spoken to since joining school, choose one, and write a script for how a conversation with them might go. In pairs, pupils bring scripts to life through role play, then repeat with different adults to explore how their communication style adapts depending on who they are speaking to.
Lesson 18: budgeting	Year 7: October	To support pupils in practicing financial decision-making around break and lunchtime in a low-pressure environment, before navigating the busy realities of the school canteen.	This activity supports pupils in learning how to manage financial decisions at break and lunch time. **“Grubs up” workbook activity:** using a school food menu with prices, pupils plan what they will eat each day for a week. Different groups are given varying amounts of money to work with, prompting discussion about meal choices, affordability, and variety. Note: practitioners should be mindful of pupils in receipt of free school meals throughout this activity.
Lesson 19: making positive decisions	Year 7: October	To help pupils distinguish between what they can and cannot control during primary-secondary school transitions, building agency, resilience, and adaptive coping strategies.	This activity will support pupils in identifying what they have control over and when they can make their own choices, and what they cannot control, and how to manage this. **Circle of control workbook activity:** in pairs, pupils discuss four transition scenarios (homework overload, new lunchtime routine, feeling anxious about making friends, getting lost) and sort factors into what is and isn’t within their control, using the Circle of Control framework. Discussion focuses on how directing energy toward controllable factors builds resilience and reduces feelings of helplessness.
Lesson 20: building bridges	Year 7: October	To develop pupils’ skills and confidence in repairing teacher-pupil relationships using a restorative approach, recognising that navigating these dynamics takes time and practice.	This activity supports pupils in developing the skills and confidence in rebuilding a teacher/pupil relationship if it has broken down. **“I’m sorry that” activity:** pupils take on different roles (themselves, each other, a teacher, a pupil) and practice introductions before progressing to role-playing conflict scenarios, such as forgetting homework, arriving late, or being caught talking. Rehearsing these difficult conversations in a safe space aims to reduce anxiety and support relationship repair.
Lesson 21: managing change and problem-solving	Year 7: October	To help pupils reflect on the changes they have navigated during transition and draw on their own experiences to develop future problem-solving strategies.	This activity will help pupils to reflect on the changes they have navigated over primary-secondary school transitions and how they have managed these changes to help future problem-solving. **“Challenge, Solution, Still to solve” workbook activity:** in groups, pupils create a three-column sheet and take turns adding transition challenges, then collaboratively discuss and complete the solution and still-to-solve columns. The activity helps pupils recognise and celebrate the progress they have already made in adapting to secondary school.

Each primary school *#TaST 5-7* lesson lasts approximately 40 min and each secondary school lesson 20 min. All lessons includes links to the RSHE curriculum objectives, important vocabulary, a warm-up activity, Forum Theater Activity and written and/or discussion based activities. Children receive a workbook, and teachers use lesson plans.

**FIGURE 2:**
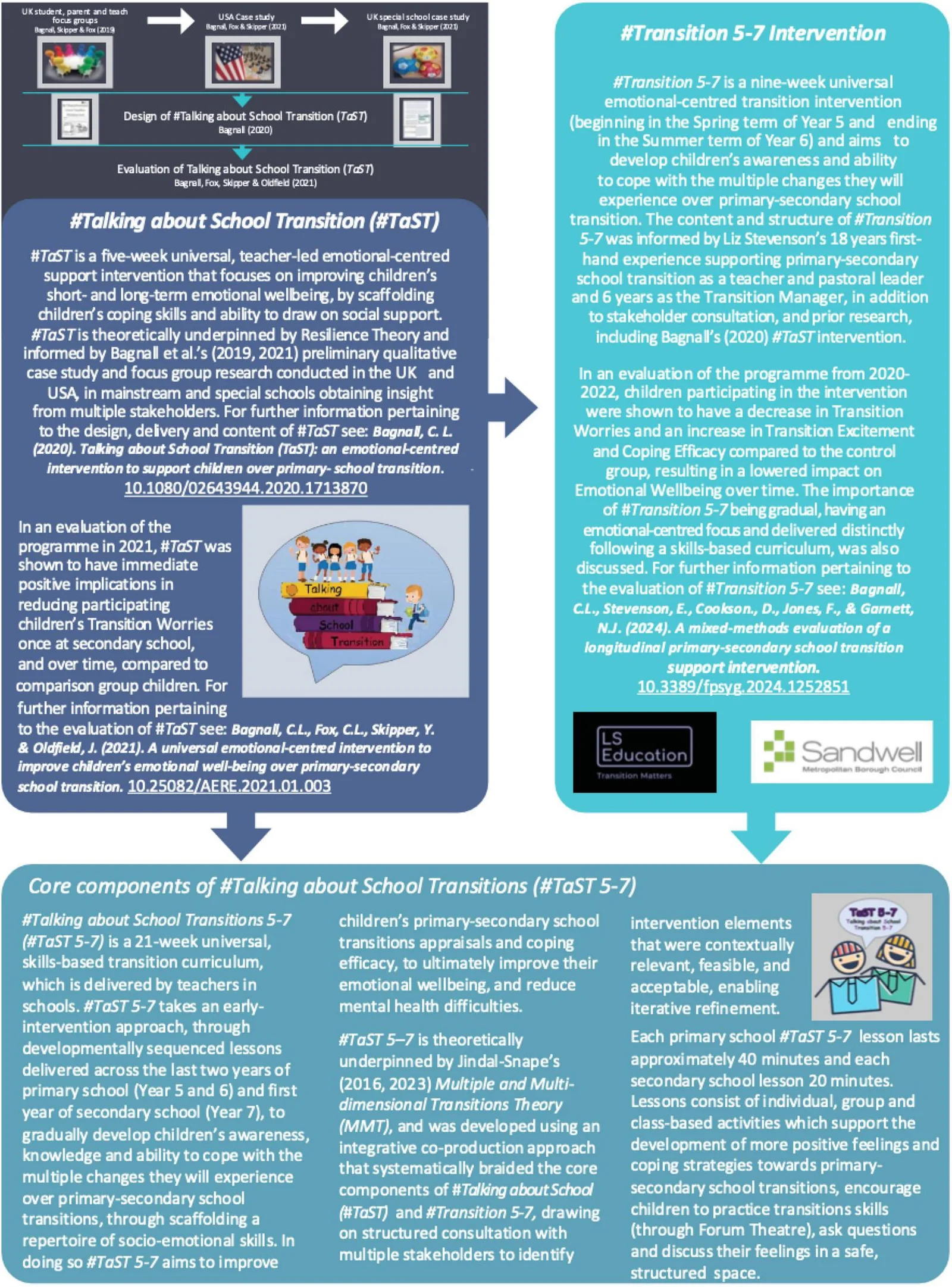
An outline of how #TaST 5-7 was developed using an integrative co-production approach that systematically braided the core components of two established primary–secondary school transition interventions. This work is licensed under the Creative Commons Attribution-NonCommercial- NoDerivatives 4.0 International License. To view a copy of this license visit: www.creativecommons.org/licenses/by-nc-nd/4.0.

*#Talking about School Transitions 5-7* is theoretically underpinned by Jindal-Snape’s (2023) *Multiple and Multi-Dimensional Transitions* (*MMT*) theory, significant research, stakeholder consultation and was developed using an integrative co-production approach that systematically braided the core components of two established primary–secondary school transition interventions (see Figure 2). This included *Talking about School Transition* (*TaST*), a 5-weeks emotional- centered intervention that focuses on supporting children’s emotional wellbeing in Year 6 leading up to primary-secondary school transitions, by scaffolding children’s coping skills and ability to draw on social support from parents/guardians, teachers and classmates (Bagnall, 2020); and *Transition 5–7*, a 9-weeks universal intervention, delivered from the Spring term of Year 5 to the Summer term of Year 6, which aims to develop children’s awareness and ability to cope with the multiple changes children experience over primary-secondary school transitions, through a Forum Theater skills-based curriculum. These small developer-led trials (Bagnall et al., 2021a,2024a) of the content, foci and delivery, which underpinned *#TaST 5-7*, have demonstrated feasibility, acceptability, utility, cost-effectiveness and reach as outlined in Table 2.

**TABLE 2:** #Talking about School Transitions 5-7 (#TaST 5-7) small developer trial outcomes.

Feasibility	The trials have been implemented as planned to date with 707 Year 5–7 children in 13 schools in England;
Acceptability	Children and teachers have reported the emotional-centered skills-based: “it showed you how to cope in different situations, and helped me to be more prepared” (Bagnall et al., 2021a, p.121 [Year 6 pupil]), and gradual early-intervention approach: “These are mindset changes for the children, these are skill changes that need to come to form habits which take time” (Bagnall et al., 2024a, p.11 [Year 6 Teacher]) to be useful;
Utility	Evidence has shown that children participating in the Year 5 and 6 components of the trials to show a decrease in transition worries and an increase in transition excitement and coping efficacy compared to the comparison group from Year 5 to Year 6, resulting in a lowered impact on emotional wellbeing in Year 7. • Cost-effectiveness: These effects were more pronounced for children with SEND, those eligible for PPF and children who are academically behind.
Reach	National support for *TaST* as a “promising universal school-based intervention that could influence policy and practice nationally” has been referenced in Department for Education (2026), National Institute of Health and Care Excellence (2022) and Health Scotland (2022) guidelines, and internationally there has been appetite by the DfE in Australia: *“#TaST reduced children’s anxiety, increased their awareness about transition and ultimately gave them the language to feel more comfortable and has transformed [named state] approach to supporting primary-secondary school transition” [Mental Health Practitioner]*, indicating strong potential for cultural transferability (Mundy, 2024) an area that has received scant attention.

Building on the positive outcomes of the small developer-led trials (Bagnall et al., 2021a,2024a), intervention integration presents clear advantages over designing programmes independently. Integration allows for perseveration of empirically validated active ingredients, minimises the risk of diluting effective components, and leverages complementary strengths across approaches (e.g., in the context of *#TaST 5-7* the combination of the *#TaST* intervention theoretical depth and *#Transition 5-7* practice-based expertise). This integrative approach enhances conceptual coherence, optimises resource utilisation by consolidating overlapping functions, and increases confidence in scalability, given that both constituent interventions have demonstrated feasibility, acceptability, utility and reach in real-world contexts. Consistent with established guidance on adapting and integrating complex interventions (Movsisyan et al., 2019), a detailed component analysis of the theoretical foundations, core components, and mechanisms of change across *#TaST* and *#Transition 5–7* was undertaken (see Table 3). This analysis drew on the process and outcome evaluation data to identify which elements of each intervention were most effective, the timing of their effects, how and why (e.g., implementation variability, intervention change mechanisms).

**TABLE 3:** *#TaST 5-7* component analysis and co-production process.

Intervention	Author & year developed	Core components	Underpinning research & theory	Stakeholder consultation and co-design process
*#Talking about School Transition* (#*TaST*)	Bagnall (2020)	#*TaST* focusses on supporting children’s emotional wellbeing over primary-secondary school transition, by scaffolding children’s coping skills and ability to draw on social support from parents/carers, peers and teachers. #*TaST* is a five week, universal emotional-centred intervention that is delivered in class by Year 6 teachers in the summer term of primary school. Intervention lessons last approximately 1-hour, are delivered on a weekly basis, through individual, pair, group and whole class spoken and written emotional expression activities. Lessons have three main foci: (1) Helping children to position the transition as a progression as opposed to a loss; (2) Building children’s coping skills and resilience; (3) Emphasising the importance of social support, how this may change at secondary school, and how to access it.	The intervention foci and structure is underpinned by Resilience Theory, particularly five background concepts that underpin the concept: a) reducing stockpile of problems, b) pathways and turning points in development, c) having a sense of a secure base, d) self-esteem and e) self-efficacy; in addition to a thorough literature review of the social, academic, environment and emotional changes negotiated during this time (Bagnall, 2020).	The core components of #*TaST* was informed by the following stakeholder consultation, which drew on children’s, educational practitioners, and parents’/carers’ experiences of primary-secondary school transitions and how this period could be improved: • Bagnall, C. L., Skipper, Y., & Fox, C. L. (2020). ‘You’re in this world now’: Students’, teachers’, and parents’ experiences of school transition and how they feel it can be improved. *British Journal of Educational Psychology*, *90*(1), 206–226. 10.1111/bjep.12273 • Bagnall, C. L., Fox, C. L., & Skipper, Y. (2021c). When is the ‘optimal’ time for school transition? An insight into provision in the US. *Pastoral Care in Education*, 1-29. 10.1080/02643944.2020. 1855669 • Bagnall, C. L., Fox, C. L., & Skipper, Y. (2021b). What emotional-centred challenges do children attending special schools face over primary–secondary school transition? *Journal of Research in Special Educational Needs*, *21*(2), 156–167. 10.1111/1471-3802.12507 Children, practitioners, and families were not simply consulted as informants but were active collaborators whose knowledge and priorities directly shaped the intervention’s design and delivery. A detailed account of how this co-produced knowledge was translated into *#TaST* is outlined here: • Bagnall, C. L. (2020). Talking about School Transition (TaST): an emotional centred intervention to support children over primary-secondary school transition. *Pastoral Care in Education*, *38*(2), 116-137. 10.1080/02643944.2020.1713870
#*Transition 5–7*		*#Transitions 5–7* aims to develop children’s awareness and ability to cope with the multiple changes they will experience over primary–secondary school transitions. *#Transitions 5–7* is a nine-week universal emotional-centred transitions intervention, which starts in the spring term of Year 5, and continues into the summer term of Year 6. Each of the nine lessons last approximately 50 minutes and are delivered on a weekly basis through individual, pair, group and whole class Forum Theatre activities.	The content and structure of #*Transitions 5–7* was designed by the Transitions Manager of the local government education authority and drew on her 18 years of first-hand experience supporting primary–secondary school transitions, in addition to previous research, including the design, implementation and evaluation of Bagnall’s (2020) *#TaST* intervention.	The core components of #*Transitions 5–7* were co-produced through meaningful partnership with children and educational practitioners. This collaborative process drew on stakeholder consultation of common primary-secondary school transitions challenges and was further supported by Bagnall’s (2020) *#TaST* intervention process evaluation findings: • Bagnall, C. L., Fox, C. L., Skipper, Y., & Oldfield, J. (2021a). Evaluating a universal emotional-centred intervention to improve children’s emotional well-being over primary-secondary school transition. *Advances in Educational Research and Evaluation*, *2*(1), 113–126. 10.25082/ AERE.2021.01.003 Following synthesis and mapping of this stakeholder consultation, #*Transitions 5–7* lessons were refined through iterative co-design with Year 5 and 6 children and teachers, ensuring that the final materials were contextually relevant and authentically reflected their needs and priorities before implementation. Amongst other decisions, this co-design process informed the decision to incorporate Forum Theatre activities to enable children to test-out learnt transition skills.
**Intervention**	**References**	**Core components**	**Underpinning research and theory**	**Stakeholder consultation and co-design process**
*#*Talking about School Transitions 5-7 (*#TaST 5-7*)		*#TaST 5-7* aims to improve children’s primary-secondary school transitions appraisals and coping efficacy, to ultimately improve their emotional wellbeing, and reduce mental health difficulties. *#TaST 5-7* is a 21-week universal, skills-based transition curriculum, which is delivered by teachers in schools. *#TaST 5-7* takes an early-intervention approach, through developmentally sequenced lessons delivered across the last two years of primary school (Year 5 and 6) and first year of secondary school (Year 7), to gradually develop children’s awareness, knowledge and ability to cope with the multiple changes they will experience over primary-secondary school transitions. Each primary school *#TaST 5-7* lesson lasts approximately 40 minutes and each secondary school lesson 20 minutes. Lessons consist of individual, group and class-based activities which support the development of more positive feelings and coping strategies towards primary-secondary school transitions, encourage children to practice transitions skills (through Forum Theatre), ask questions and discuss their feelings in a safe, structured space.	*#TaST 5-7* is theoretically underpinned by Jindal-Snape’s (2023) *Multiple and Multi-Dimensional Transitions (MMT)* theory, and significant research, including Beatson et al.’s (2023) systematic literature review which synthesised and evaluated evidence to date pertaining to the design, selection, implementation and evaluation of primary-secondary transition interventions.	#*TaST 5–7* was co-developed using an integrative co-production approach that systematically braided the core components, and stakeholder consultation which underpinned Bagnall’s (2020) *#TaST* and Bagnall et al.’s (2024b) #*Transitions 5–7* interventions. This included the developer-led trials of the content, foci and delivery, which underpinned *#TaST 5-7*: • Bagnall, C. L., Fox, C. L., Skipper, Y., & Oldfield, J. (2021a). Evaluating a universal emotional-centred intervention to improve children’s emotional well-being over primary-secondary school transition. *Advances in Educational Research and Evaluation*, *2*(1), 113–126. 10.25082/ AERE.2021.01.003 • Bagnall, C.L., Cookson, D., Stevenson, L., Jones, F. & Garnett, N.J. (2024a). Evaluation of a longitudinal transition support intervention to improve children’s emotional well-being and adjustment over primary-secondary school transition. *Frontiers Educational Psychology.* 10.3389/fpsyg.2024.1252851 Following synthesis and mapping of this stakeholder consultation, #*TaST 5–7* lessons were refined through iterative co-design with Year 5, 6 and 7 children and teachers. Amongst other decisions, through this co-design, it was decided that the Year 7 lessons should be separated into 4 X 20-minute form time activities, as opposed to 8 X 40 minute PSHE lessons. Educational practitioners also thought it was important to link each primary school lesson with a RSHE objective and include a warm-up activity prior to Forum Theatre activities.

Embedding co-production within educational research is critical to bridge gaps between research, policy, and practice, ensuring that innovation is both evidence-informed and practice-led. In the development of *#TaST 5–7*, stakeholders (e.g., pupils, teachers, parents) engaged in structured consultation to identify intervention elements that were contextually relevant, feasible, and acceptable, enabling iterative refinement. Practitioners contributed essential contextual expertise regarding children’s needs, school cultures, and systemic constraints. This collaborative approach enhanced relevance and feasibility, while fostering practitioner ownership and professional empowerment (Schildkamp, 2019).

In sum, through the systematic braiding of two evidence-informed interventions, *#TaST 5–7* generates innovation grounded in established mechanisms of change, indicative of rigorous component analysis, and iterative stakeholder consultation, to maximise effectiveness, sustainability, and transferability across diverse school settings. Outlined is a detailed account of the key components underpinning the conceptualisation, design and implementation of *#TaST 5–7*, drawing on established intervention science guidelines (Humphrey et al., 2016). This includes the methodological decisions underpinning development, which was grounded in prior research, theory and practice-based insights, to enhance transparency around design, adaptation, implementation, and evaluation processes for researchers and practitioners seeking to develop, refine, or apply interventions within their own contexts. Throughout best-practice guidance pertaining to supporting children’s emotional wellbeing during primary–secondary transitions will be made, to improve knowledge and understanding, and the application of evidence-informed strategies.

### Design

#### Emotional wellbeing foci

As shown in Beatson et al.’s (2023) systematic literature review, transitions interventions are limited in their focus on supporting children’s emotional wellbeing over primary–secondary school transitions, with most interventions centered around the practicalities of school transitions, preparing children for the new ways of learning, and social changes (Jindal-Snape et al., 2020; White, 2020). Recognising that for up to 2/3 of children report primary-secondary school transitions to be emotionally demanding, and heavily draw on their ability to cope, there is need to take a preventative as opposed to curative approach through intervention focused on supporting children’s emotional wellbeing during this time, which *#TaST 5–7* does. In line with this, the intervention foci and structure is not only theoretically underpinned by the five tenants of Jindal-Snape’s (2023) *Multiple and Multi-Dimensional Transitions*, but also expands this theoretical framework to consider children’s emotional appraisals in the context of managing the cognitive, behavioral, and emotional demands of primary-secondary school transitions. This concept represents the new emotions that children must manage in response to novel experiences navigated during this period, and highlights that, without this contextual element, emotional wellbeing as a general construct does not fully capture children’s lived experiences and feelings managing primary–secondary transitions (Bagnall et al., 2025a). In addressing this gap, *#TaST 5–7* makes a novel theoretical contribution to knowledge in addressing the lack of conceptual clarity and operationalisation in how we define children’s emotional wellbeing in the context of primary-secondary school transitions to ultimately intervene, through a structured, theory-informed intervention, that has been informed by children’s own conceptualisations.

Children’s voices are chronically underrepresented within the field, with less than 1/3 of research studies drawing on children’s perspectives (Jindal-Snape et al., 2020). However, children are, without question, the most knowledgeable informants of their own emotional wellbeing in the context of their primary-secondary school transitions experiences. Thus, integrating their voice alongside those of teachers and caregivers ensures that the transitions process is conceptualised in a holistic way (Packer and Pierce, 2025), which was integral to the development of *#TaST 5–7*. For example, an integral attribute of emotional wellbeing in the context of primary-secondary school transitions include children’s affective experience of navigating primary-secondary school transitions in the here-and-now. *#TaST 5–7* targets children’s in the moment affective experiences through scaffolding children’s awareness, understanding and confidence in handling the immediate emotional demands of primary-secondary school transitions. Recognising the dual nature of primary-secondary school transitions, combining both a sense of optimism and anticipation with anxiety and fear, *#TaST 5–7* encourages children through the guided activities to ask questions, discuss their feelings, and reflect on their emotional responses, within a supportive, safe environment. This was discussed as paramount within the *#TaST* intervention in helping children to understand and manage uncomfortable in the moment feelings: “helped us understand the worries about high school” and “stopped me worrying,” through a shared, supportive experience: “it helped me know that I was not alone” (Bagnall et al., 2021a, p.120).

#### External resources: social support

Underpinning children’s and educators’ conceptualisation of emotional wellbeing in the context of primary-secondary school transitions is children’s perceptions of their internal and external resources to manage the demands of primary-secondary school transitions and maintain a stable affective state. Embedded within the *#TaST 5–7* lessons, children are encouraged to reflect and draw on support from peers, teachers, and parents/guardians, through guided activities. For example, integral to the group and class-based activities delivered through Forum Theater, is scaffolding peer support, and encouraging children to recognise primary-secondary school transitions as a shared experience, fostering a sense of collective understanding and emotional containment (Jindal-Snape and Miller, 2008). Similarly, lessons incorporate reflection on teacher support, highlighting the complementary roles of primary and secondary teachers in preparing children for secondary school and helping them settle into secondary school (Hopwood et al., 2016; Coffey, 2013), while class-based activities address realistic expectations around changes in teacher relationships and strategies for accessing support.

Structured home-learning activities play a critical role in extending support beyond the classroom to parents/carers, such as the child-led parent puzzler activity, which scaffolds supportive and sensitive discussions between children and parents/carers relating to primary-secondary school transitions; thereby extending support and reinforcing adaptive coping and reflection. For example, for this activity children jot down eight questions they would like to ask their parents about primary-secondary school transitions (e.g., What was your first day like?; Did you get lost?; What were the school lunches like?) in their puzzle. Using the puzzle template, the child then asks their parents these questions, to guide open communication and sensitive discussion around primary-secondary school transitions. One helpful component of this activity is that it is child-led, in that children select the topics that they would like to talk to their parents about. This is important to ensure that discussions are sensitive and guided by children’s window of tolerance, as outlined in previous research: “If they make too much of a fuss about it then it does proper worry you, it’s like, like a soldier preparing for war like, if they give them a whole entire suit of armor it’s then they can think, what are we going against.” (Year 7 pupil; Bagnall et al., 2020, p. 13).

#### Internal resources: coping efficacy

Consistent research within the field has shown coping efficacy (e.g., children’s perceived capability and belief in their ability to cope) to be more predictive of successful primary-secondary school transitions adjustment than coping skills (Bagnall et al., 2021a; Jindal-Snape and Cantali, 2019; West et al., 2010). This is consistent with Bandura’s (1997) self-efficacy theory, and Lazarus and Folkman’s (1984) transactional model of stress, both of which highlight the central role of perceived capability in shaping responses to challenge. Together, these frameworks suggest that coping efficacy influences secondary appraisal processes, e.g., when a child believes they can manage a situation, it is less likely to be experienced as overwhelming, even prior to the enactment of specific coping strategies. In turn, this perceived capability determines whether and how coping strategies are mobilised during periods of stress. Drawing on this theoretical and empirical insight, *#TaST 5–7* explicitly focuses on developing children’s coping efficacy through structured Forum-Theater activities which scaffold children’s mastery in navigating common secondary school challenges (which have been informed by significant stakeholder consultation), such as rehearsing how to approach new teachers, problem-solve common peer dilemmas and navigate a school timetable and map. This is recognising that intervention activities linked to children’s real-life lived experiences help children to provide meaning to future events and this has been shown in the context of primary-secondary school transitions, with children discussing the usefulness of being able to “test-out” learnt transitions strategies, through Forum Theater, alongside their classmates, to develop their skills in being able to manage discontinuity: “I think less talking and more action, like get people to interact with the activities because it’s good to feel it like what you are actually doing, what it will be like at high school” (Year 6 pupil; Bagnall et al., 2024a, p.12).

Integral to these activities is guided reflection, to normalise feelings of discomfort when navigating novelty and uncertainty, while strengthening children’s coping efficacy by reinforcing their agency, growing competence and self-belief, which can take time: “These are mindset changes that need to come from the children, these are skill changes that need to come and habits do not change overnight and there needs to be that long term preparation for them, and actually, then you are not even forming the habit until they get to secondary school” (Programme developer; Bagnall et al., 2024a, p.12). As part of this *#TaST 5–7* encourages children to reflect on previous transitions they may have experienced, such as moving house, how they felt during this time, what skills they learnt and how they could translate those skills to primary-secondary school transitions; this is in recognition of previous research which has shown children who have been exposed to previous transitions experiences to find primary-secondary school transitions easier, often as they have developed coping beliefs, skills and experiences which form as templates for future transitions (Adeyemo, 2005; Bagnall et al., 2021c).

#### Early-intervention, contiguous approach

**FIGURE 3:**
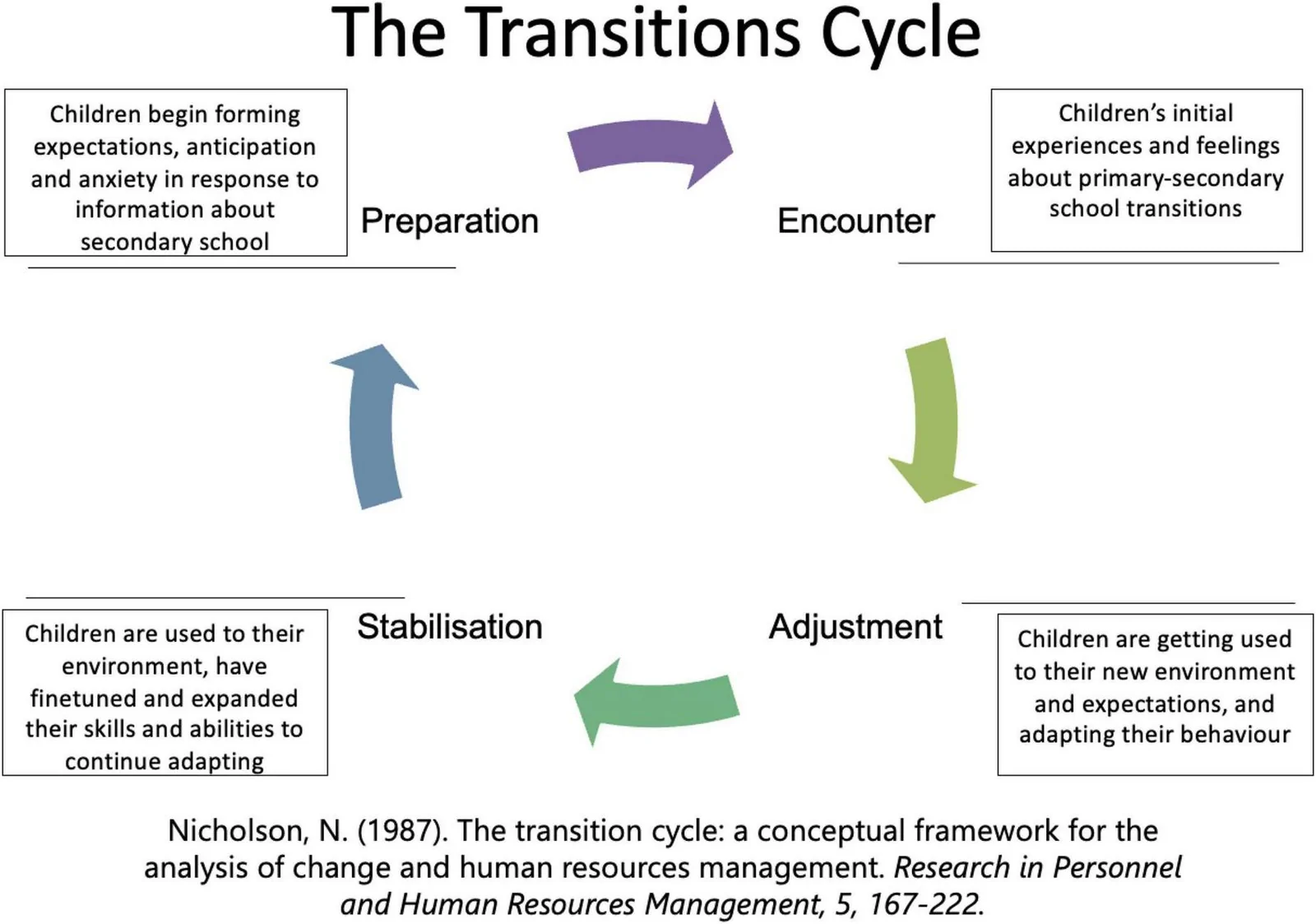
Based on Nicholson’s (1990) transitions cycle, adapted to the context of primary-secondary school transitions (Bagnall and Jindal-Snape, 2025). This work is licensed under the Creative Commons Attribution-NonCommercial-NoDerivatives 4.0 International License. To view a copy of this license visit: www.creativecommons.org/licenses/by-nc-nd/4.0.

There is a dearth of longitudinal research on primary–secondary school transitions, and this methodological limitation extends to intervention research, where there are no interventions to date that adopt an early-intervention, preventative approach beginning in Year 5, combined with a contiguous structure into the first two terms of Year 7. #*TaST 5–7* addresses this gap by providing a coordinated, cross-phase curriculum, that gradually develops children’s awareness, knowledge and ability to cope with the multiple changes children encounter over primary-secondary school transitions. Underpinned by principles of adaptability (Holliman et al., 2022) and aligning with Nicholson’s (1990) transitions cycle, which we have adapted in the context of primary-secondary school transitions (see Figure 3), #*TaST 5–7* lessons take a “non-threatening” approach that gradually supports children in managing the cognitive, behavioral, and emotional demands of primary-secondary school transitions. In Year 5, the curriculum initially focuses on the development of transferable skills, such as asking for help, and making decisions, which children are likely to have experienced in situations of novelty and uncertainty in their day-to-day life, e.g., asking for help in finding grocery’s in a supermarket.

This foundation lays the groundwork for the more explicit primary–secondary school transitions content explored in Year 6, where attention shifts to exploring similarities and differences between primary and secondary school to understand upcoming changes, before incrementally contextualising learnt skills in regulating thinking, behavior, and emotions when facing uncertainty in relation to typical primary-secondary school transitions scenarios. This gradual, progressive approach embodies the principles of adaptability, scaffolding children’s exposure and engagement with primary-secondary school transitions changes incrementally to support preparedness while minimising feelings of overwhelm. This aligns with previous research which has shown primary-secondary school transition provision to be most effective when exposure to secondary school is gradual and scaffolded, providing children with honest insight into what to expect and how to navigate differing expectations, while maintaining a degree of consistency, as too much, too soon can lead to feelings of overwhelm and anxiety (Bagnall et al., 2020).

This approach also prevents feelings of rumination, as prior to entering the preparation phase of the transitions cycle, which is when children begin forming expectations, anticipation and anxiety in response to upcoming transitions changes, children will have already began to develop transferable skills to manage the cognitive, behavioral, and emotional demands of primary-secondary school transitions. The efficacy of this approach can be shown through the qualitative *#Transition 5-7* data where children explicitly discussed the value of this gradual, progressive approach in helping children to feel more prepared: “in Year 5, the transitions lessons were getting you prepared by practicing things for when it comes,” especially for their induction days: “We started it in Year 5 because then we could use it in Year 6 and at our induction days but if we started it straightaway in Year 6, then we would not be as prepared for our induction days” (Year 6 child; Bagnall et al., 2024a, p.11–12).

Aligning with Bagnall and Jindal-Snape’s (2025) transitions cycle, the Year 7 lessons focus on supporting encounter and adjustment processes to support stabilisation, which is when children ultimately become used to their new environment. The encounter phase represents children’s initial experiences of primary-secondary school transitions, and during this time, focus is placed on learning new routines and standards at secondary school and building connections with new peers and teachers. To support with this, the first four secondary school *#TaST 5–7* lessons, which are delivered in September (the first month of Year 7), focus on encouraging children to get to know, and navigate new people and places in their new secondary school building. This includes learning how to read a school map, recognising that for many pupils, this may be the first time that they see a map that looks like this. Practicing how to read it before it needs to be used, through this activity, can help children feel more confident and support them in navigating their way around their school building. Other lessons within this phase focus on scaffolding children’s confidence in speaking to new secondary school teachers and pupils in secondary school that they don’t know or trust yet, to support teacher and peer support. Integral to these activities is supporting children develop the skill of finding and being an appropriate version of themselves based on who they are speaking to, which supports skills such as social awareness and emotional intelligence and helps children to build positive relationships and communicate effectively in different contexts.

Entering into the adjustment phase, the final four secondary school lessons in October recognise that children are getting used to their new secondary school environment and expectations. The focus of the lessons shift toward supporting children to adapt their behavior in response to successful and unsuccessful experiences, through structured activities centered on budgeting and managing financial decisions at break and lunch time; making positive, adaptive choices by distinguishing between what they can and cannot control; and building bridges to rebuild and strengthen teacher–pupil relationships where needed. The final lesson also encourages children to reflect on the changes they have navigated over primary-secondary school transitions, to help future problem-solving.

In sum, by operating within this “goldilocks zone,” where the pace of sessions is slow enough to prevent overwhelm but frequent enough to provide meaningful exposure, *#TaST 5–7* aims to provide greater opportunities for children to engage with transition knowledge, practice skills, ask questions, and discuss their feelings, helping them feel prepared and supported.

#### Organisational readiness and practicalities of a contiguous approach over primary-secondary school transitions

Readiness for change is often conceptualised at the individual level in terms of capability, opportunity, and motivation. In the context of primary–secondary school transitions, however, effective implementation requires organisational readiness across both primary and secondary school phases, including a shared commitment to transitions as a collective responsibility and confidence in teachers’ capacity to work across institutional boundaries. From an implementation science perspective, such cross-phase readiness is critical for the sustainability and scalability of interventions, and thus central to the implementation of *#TaST 5–7* was the intentional strengthening of structured communication, collaboration, and coordination between primary and secondary schools. This was achieved through cross-phase webinars and train-the-trainer workshops, during which the core components and theoretical foundations of *#TaST 5–7* were presented and discussed. Emphasis was placed on integrating evidence from both phases demonstrating that early and sustained engagement between primary and secondary teachers can improve children’s social-emotional adjustment, confidence, and academic engagement (Bagnall et al., 2020).

Furthermore, it was clear from our developer-led trials that implementation effectiveness can be dependent on having sufficient flexibility and resources, which are not always available within schools. Thus, to overcome this, primary and secondary school educators received full lesson plans and pupil workbooks at least two months ahead of delivery, allowing ample time for familiarisation with the content and structure of *#TaST 5–7*. The *#TaST 5–7* materials were also ready-to-use, allowing teachers to deliver sessions directly from the pack with minimal preparation. This reduced workload, promoted fidelity to the intervention’s theoretical framework and core components, and supported consistent delivery across diverse school contexts, enabling teachers to focus on facilitating discussion, reflection, and pupil engagement. Furthermore, early access also facilitated planning for timetabling, coordination with colleagues across phases, and integration with existing school priorities, further supporting successful implementation and sustainability, drawing on the success of this approach within the *#Transition 5-7* pilot: “having the pre-planned timetable worked well, I’d set aside weeks, in my diary for the whole 2 years sessions, so I was never scratting for time” (Year 6 Teacher; Bagnall et al., 2024a, p.11).

## Discussion

In sum, primary-secondary school transitions are emotionally demanding, critical periods for children, which pose a heightened risk for the development of poor emotional wellbeing and subsequent mental health difficulties and academic disengagement (West et al., 2010) especially for more disadvantaged groups (Donaldson et al., 2025). Thus, from a public health perspective, timely support is crucial, which is recognised in policy, practice and research. However, significant limitations pervade across all three sectors, which are well documented (Bagnall et al., 2026; Beatson et al., 2023). Addressing this gap, this paper presents the theory, empirical research, and methodological decisions, which informed the design and implementation of *#TaST 5-7*: the first emotional-centered, skills-based intervention, that is delivered contiguously across Year 5, 6 and 7. In doing so, the present research, demonstrates the utility of taking an integrative co-production approach in the design and implementation of school-based interventions, drawing on rigorous component analysis, and iterative stakeholder consultation. This was paramount to ensure that innovation was evidence-informed and grounded in established mechanisms of change, but also practice-led to maximise effectiveness, confidence in scalability, and transferability across diverse school settings, directly tackling ongoing limitations that pervade the sector.

### Core components

*#Talking about School Transitions 5-7* aims to develop children’s transitions awareness, skills and ability to cope with the multiple changes they will experience over primary-secondary school transitions. These aims are in line with previous research which has shown that children’s adjustment over primary-secondary school transitions to be shaped by individual-level factors, such as coping efficacy (Bagnall et al., 2021a) and emotional intelligence (Nowland and Qualter, 2020), external factors such as school size, culture and ethos (Symonds and Galton, 2014) (and children’s awareness of these factors beforehand) (Bagnall et al., 2024a), and social factors such as positive, supportive relationships with parents/carers, teacher and peers (Bagnall et al., 2020). By foregrounding coping efficacy, relational support, and in-the-moment emotional appraisal within a multi-dimensional transitions framework (Jindal-Snape, 2023), this work contributes both theoretically and methodologically to transitions literature. There is dearth of research within the field exploring the causal structures and antecedents of positive outcomes within the primary-secondary school transitions context. For example, we do not know which components of programmes are most effective in predicting desired outcomes (e.g., for our programme do we see the most change in children’s emotional wellbeing following a specific number of lessons targeting children’s coping efficacy and transitions appraisals), or to what extent is parental and/or classmate support a protective factor, and how is this scaffolded within the intervention. This inhibits our understanding of what is most important within curricula to support primary-secondary school transitions and has contributed to inconsistencies within the field and fragmented “patchwork” practice across schools and Local Authorities (Bagnall et al., 2026; Growney, 2022).

Furthermore, interventions that are explicitly grounded in clearly articulated mechanisms of change are more amenable to adaptation without dilution, enhancing their sustainability and viability across diverse educational contexts. This supports cumulative knowledge building by enabling future research to test, refine, and compare the core components of transition interventions. For practitioners, specifying mechanisms provides clarity about why particular components matter, which strengthens fidelity, intentional implementation, and contextual tailoring. Collectively, these considerations suggest that future transition research should prioritise mechanism specification, transparent reporting of adaptation processes, and evaluation designs that examine not only outcomes, but also how, for whom, and under what conditions change occurs.

### Co-production

While universal school-based interventions have demonstrated effectiveness in improving mental health and emotional wellbeing, this evidence largely derives from quantitative pre–post evaluations (Mackenzie and Williams, 2018). This has contributed to the dominance of process based logic models, which theorise intervention mechanisms at a granular level, rather than system-based logic models, that more fully conceptualise the relationship between an intervention and its contextual conditions. Despite this emphasis on intervention processes, there remains little qualitative research exploring children’s own experiences participating in these interventions, and teachers’ experiences delivering them (Foulkes and Stapley, 2022). Thus, the present study addresses this gap, by embedding children’s and teachers’ perspectives through iterative co-production to ensure that the design and delivery of *#TaST 5-7* is contextually relevant, and responsive to diverse lived experiences (Ching et al., 2024; Foulkes and Stapley, 2022). Intervention modifications were guided by systematic engagement with stakeholder values, experiences and theorisation of contextual features that may interact with the way in which *#TaST 5-7* was implemented, while developers ensured fidelity to core components of *#TaST 5-7* and underpinning theory. In doing so, the study advances theory development, that has historically marginalised lived experiences (Kneale et al., 2025; Ozturk et al., 2025), especially within primary-secondary school transitions research (Jindal-Snape and Bagnall, 2023), providing a worked example of the value of co-production and a transdisciplinary perspective that integrates knowledge, methods, and perspectives from multiple stakeholders to address real-world challenges in practice.

However, efforts to support children’s emotional wellbeing over primary-secondary school transitions remains a systemic challenge (Department for Education, 2023b), with schools typically having little leverage in changing higher-level factors in the system (e.g., at the policy level). Nevertheless, the influence of these system-wide factors on children’s emotional wellbeing in the context of primary-secondary school transitions remains important to acknowledge and theorise. This tension mirrors some concerns around the “cruel optimism” of co-production, and the extent to which co-producers are shouldered with responsibilities to address problems that are beyond their remit to address (Duggan et al., 2025). In the context of the present study, there is need to formalise minimum practice standards for primary–secondary transitions for both primary and secondary schools through statutory guidance, e.g., embed transitions planning and support within school improvement and pastoral care frameworks, and/or include transitions quality indicators in Ofsted criteria focused on wellbeing, inclusion, and transitions preparedness, as outlined in the National Primary-Secondary School Transitions Strategy (Bagnall et al., 2026). Given the time and resource pressures schools face, this would provide a first step in ensuring effective and equitable transitions provision, by providing a consistent framework, incorporating measurable objectives, quality markers, and structured support drawing on high-quality, evidence-informed resources and programmes, such as *#TaST 5-7*, to help reduce disparities in provision across schools and regions (Evangelou et al., 2008).

### Continuity

Despite the recognised importance of cross-phase collaboration over primary-secondary school transition, research consistently highlights a lack of structured communication, collaboration and coordination between primary and secondary schools (Mumford and Birchwood, 2021). This gap can result in fragmented transitions experiences, poor information sharing, misaligned expectations, and limited continuity in both academic and socio-emotional support (Bagnall et al., 2026; Evangelou et al., 2008). Research also suggests that early engagement and sustained collaboration between primary and secondary teachers can improve transition outcomes, including children’s social-emotional adjustment, confidence, and academic engagement (Bagnall et al., 2020). Thus, *#TaST 5–7* makes a first step in demonstrating the effectiveness of a joint transitions curriculum, that combines early intervention with cross-phase continuity, and a common language that has been co-produced with primary and secondary teachers to ensure alignment across phases, foster teacher engagement, and enhance feasibility, sustainability, and reach. In doing so, this curriculum helped facilitate greater awareness and understanding between primary and secondary phases through direct engagement with shared resources, thereby strengthening continuity between schools. These findings underscore the need to position school transitions as a collective responsibility and to foster confidence in teachers’ capacity to collaborate effectively across institutional boundaries.

However, to do this, there is need for coordinated, system-wide action over primary-secondary school transitions. In part, challenges may stem from inadequate conceptualisation of transitions as a one-off “event,” as opposed to an ongoing process of adaptation across domains and contexts (Jindal-Snape, 2016, 2023). This narrow framing has contributed to the development of policies and practices that are not fit for purpose (Hannah et al., 2023; Jindal-Snape et al., 2020; Symonds et al., 2023). For instance, considering primary-secondary school transitions in line with the former, support could involve a single visit or handover meeting, rather than as a continuous, planned process encompassing curriculum alignment, pastoral support, and structured parent/carer communication and engagement (Jindal-Snape, 2023). The same applies in relation to the design and operationalisation of interventions, which to date have been temporary and short-term, predominantly delivered in the last few months of primary school, as opposed to taking a longitudinal approach. Such short-term approaches have resulted in suboptimal transitions policies and practices (Bagnall and Jindal-Snape, 2023). Therefore, it is essential that policymakers and practitioners critically engage with research literature and work collaboratively with researchers through co-production to ensure that transitions policies and practices are both evidence-informed and grounded in real-world practice. This underscores the importance of ongoing professional development and research capacity building to strengthen the reciprocal flow of knowledge between research, policy, and practice. It also highlights the responsibility of researchers to disseminate their research in ways that are accessible to different stakeholders.

### Limitations

Concerns have been raised regarding the time and resource demands associated with deep co-production in research. The development of *#TaST 5–7* was a substantial undertaking, involving mapping the design, implementation and evaluation of two distinct interventions, that were then systematically braided, alongside the iterative co-production of an overarching logic model. From an implementation science perspective, this investment was necessary to enhance contextual fit, acceptability, and feasibility across primary and secondary settings; factors consistently associated with implementation quality, sustainability and reach. Critically, the co-production process enabled careful navigation of the fidelity–adaptation tension: adaptations were systematically informed by stakeholder perspectives to improve contextual relevance, while core components and theoretical mechanisms were safeguarded to maintain intervention integrity. Grounded in participatory research principles, co-production was therefore not treated as an optional methodological enhancement but as a commitment to inclusivity, shared decision-making, and responsiveness to lived experience.

It is also important to recognise that an intervention cannot be fully understood without examining its implementation. Evaluating how adaptations were enacted, the influence of context, and the ways in which stakeholders engaged with the intervention represents the next critical phase of this programme of research. Consistent with early-phase intervention development literature (Humphrey et al., 2016), it is important to acknowledge that this paper is preliminary in nature, focusing on the theory, empirical research, and methodological decisions, informing the design and implementation of *#TaST 5-7*, rather than the evaluation of its outcomes, and should be interpreted accordingly. Such evaluation will ensure that both outcomes and processes inform the ongoing development of effective, contextually responsive support for children during this critical period. Following this, replication of evidence-informed interventions across educational settings is then needed to identify gold-standard support for children’s emotional wellbeing.

## Conclusion

In sum, the design and implementation of *#TaST 5–7* addresses systemic challenges identified within primary-secondary school transitions research, policy and practice. Responding to longstanding calls to strengthen conceptual clarity, methodological decision making and ecological fit within the design and implementation of school-based intervention, the present research has demonstrated the utility of integrative co-production, drawing on rigorous component analysis (which to date has been neglected in this context) and iterative stakeholder consultation to understand how to best support children’s emotional wellbeing through contiguous primary-secondary school transitions intervention. This was paramount to ensure that innovation was evidence-informed and grounded in established mechanisms of change, but also practice-led to maximise effectiveness, confidence in scalability, and transferability across diverse school settings, directly tackling ongoing limitations that pervade the sector. In doing so the present study has provided a conceptually coherent and scalable framework to inform further researchers and practitioners seeking to develop, refine, or apply interventions within their own contexts. Furthermore, in presenting the theory and empirical research underpinning *#TaST 5–7*, the present research has practical utility in equipping educators with the knowledge to understand children’s emotional needs and apply evidence-informed strategies to support their emotional wellbeing during primary–secondary transitions in practice.

## Data Availability

Data is available on request, as still being analysed. Requests to access these datasets should be directed to CB, charlotte.bagnall@manchester.ac.uk.
